# Correction to: Improving the usability and comprehensiveness of microbial databases

**DOI:** 10.1186/s12915-020-00831-2

**Published:** 2020-07-28

**Authors:** Caitlin Loeffler, Aaron Karlsberg, Lana S. Martin, Eleazar Eskin, David Koslicki, Serghei Mangul

**Affiliations:** 1 Department of Computer Science, University of California Los Angeles, 404 Westwood Plaza, Los Angeles, CA 90095 USA; 2 Department of Clinical Pharmacy, University of Southern California School of Pharmacy, 1985 Zonal Ave, Los Angeles, CA 90089 USA; 3 Department of Human Genetics, David Geffen School of Medicine at UCLA, 695 Charles E. Young Drive South, Box 708822, Los Angeles, CA 90095 USA; 4 Department of Computational Medicine, David Geffen School of Medicine at UCLA, 73-235 CHS, Los Angeles, CA 90095 USA; 5 School of Computer Science and Engineering, The Pennsylvania State University, 207 Electrical Engineering West, University Park, State College, PA 16802 USA; 6 Department of Biology, The Pennsylvania State University, 208 Curtin Rd, University Park, PA 16802 USA; 7 The Huck Institutes of the Life Sciences, The Pennsylvania State University, 101 Huck Life Sciences Building, University Park, PA 16802 USA

**Correction to: BMC Biol (2020) 18:37**

**https://doi.org/10.1186/s12915-020-0756-z**

Following publication of the original article [1], the authors identified an error in the affiliations of Serghei Mangul: the second affiliation (number 8) needs to be removed. The corrected affiliation information for Serghei Mangul can be found in the affiliation list of this Correction article.
